# Estimating the Social Visibility of Abortions in Uganda and Ethiopia Using the Game of Contacts

**DOI:** 10.1111/sifp.12278

**Published:** 2024-11-12

**Authors:** Margaret Giorgio, Solomon Shiferaw, Fredrick Makumbi, Assefa Seme, Simon Peter Sebina Kibira, Sarah Nabukeera, Selena Anjur‐Dietrich, Mahari Yihdego, Niguse Tadele, Elizabeth Sully

**Affiliations:** ^1^ Guttmacher Institute New York NY 10038 USA; ^2^ Department of Reproductive Health and Health Service Management, School of Public Health Addis Ababa University Addis Ababa Ethiopia; ^3^ School of Public Health Makerere University Kampala Uganda; ^4^ Bloomberg School of Public Health Johns Hopkins University Baltimore MD 21218 USA

## Abstract

Social network–based data collection methods that rely on third‐party reporting have emerged as a promising approach for measuring abortion in restrictive settings. In order for these methods to accurately measure abortion incidence, they must also assess the visibility of abortions within social networks. Failure to do so may result in estimates affected by transmission bias, caused by imperfect knowledge of all abortions within one's social network. In this paper, we present exploratory research that uses respondent‐driven sampling (RDS) and the game of contacts method to measure abortion visibility in four sites in Uganda and Ethiopia. We assess the existence of potential biases in the game of contacts estimate of abortion visibility in each site by conducting several internal and external validity tests. While these tests provided some promising results, other factors such as the representativeness of the RDS samples, direct versus indirect abortion knowledge transfers, and the generalizability of the study sites may have introduced biases into the final estimates of abortion visibility in this study. We conclude by making recommendations on how applications of this methodology could be improved to better estimate abortion‐related transmission bias.

## INTRODUCTION

Accurate estimates of the incidence of induced abortion are essential for understanding sexual and reproductive health outcomes in a population. Enumerating the number of induced abortions is necessary to accurately estimate the prevalence of unintended pregnancy. In addition, these estimates can highlight a need for safe abortion services and provide important insights into the use and provision of contraceptives. However, currently, available methods for estimating abortion incidence in legally restrictive settings suffer from several limitations. In these settings, women often have abortions outside of the formal health care system, leaving official records incomplete and thus inaccurate (Singh et al. 2018), and measuring induced abortion directly in surveys often produces underestimates due to social desirability response bias (R. K. Jones and Kost 2007; Lindberg et al. 2020; E. F. Jones and Forrest 1992).

As a result of these challenges in directly collecting information on abortions, women who induce abortion in restrictive settings are often referred to as a “hidden population,” meaning that no sampling frames for this group exist. As such, researchers have relied on indirect methods to estimate the incidence of induced abortions and the circumstances under which they occur. The abortion incidence complications method (AICM) is a commonly used indirect measurement technique(Singh, Prada, and Juarez 2010). This method measures one of the rarely visible aspects of abortion where it is legally restricted—the receipt of postabortion care (PAC) in the formal health system—which is later combined with expert's knowledge of the proportion of all abortions that results in PAC to estimate the total number of induced abortions in a study population. However, in recent years, increased access to medication abortion has likely reduced the number of women seeking treatment for complications after their abortions (Footman et al. 2018; Moseson et al. 2020), which may be diminishing the reliability of the AICM over time. As such, there is a critical need to develop new methods to accurately estimate the incidence of induced abortion in legally restrictive settings.

In response, a growing body of research is exploring the use of indirect methods that rely on individuals’ social networks and third‐party reporting (TPR) to estimate the incidence of induced abortion (Sully, Giorgio, and Anjur‐Dietrich 2020; Bell et al. 2020; Rossier 2010; Giorgio, Sully, and Chiu 2021; Yeatman and Trinitapoli 2011). One distinguishing factor of these methods is how each one defines the underlying social network. For example, some methods focus on smaller social networks made up of “strong” or close social ties, such as someone's best friend or their closest two or three confidantes (Bell et al. 2020; Sedgh and Keogh 2019; Yeatman and Trinitapoli 2011). Others use a broader definition that also includes “weak” social ties, for example, anyone in a person's broader social circle that the person has been in contact with in the past year (Rossier 2010; Sully, Giorgio, and Anjur‐Dietrich 2020). While applications of these methods have highlighted some promising features of TPR approaches and avenues for future research, they have also highlighted the existence of substantial biases that impact the validity of the resulting incidence estimates (Giorgio, Sully, and Chiu 2021; Owolabi et al. 2023; Rossier 2010; Sully, Giorgio, and Anjur‐Dietrich 2020).

A major driver of these biases is that each method has been shown to violate at least one of the general assumptions that must be met in order for social network–based methods to produce unbiased abortion estimates. The first assumption is that the method must be able to generate a sample from respondents’ social networks that is generalizable to the underlying target population of interest. For example, if the goal of the research is to estimate abortion incidence among women, aged 15–49 in Kenya, respondents’ social networks (however, defined by the specific TPR method) must, on average, be representative of all women aged 15–49 in Kenya. Previous research suggests that methods that rely on social networks made up of “strong” or close social ties (i.e., limiting the social network to a person's closest confidantes or best friend) produce biased samples that do not represent the underlying target population of women of reproductive age, resulting in unreliable estimates of induced abortion (Giorgio, Sully, and Chiu 2021). In contrast, studies have demonstrated that the network scale‐up method (NSUM), which uses broad definitions of social networks (i.e., strong and weak social ties), can generate sufficiently generalizable samples from respondent's social networks in varied settings (Sully, Giorgio, and Anjur‐Dietrich 2020; Feehan and Salganik 2016; Guo et al. 2013; Salganik, Fazito, et al. 2011; RBC/IHDPC 2012). As such, researchers have argued that the use of broad social network definitions and the NSUM approach is a more promising avenue for estimating abortion incidence using social network–based TPR methods (Giorgio, Sully, and Chiu 2021; Sully, Giorgio, and Anjur‐Dietrich 2020).

The second assumption shared by social network–based TPR methods is that respondents are able to provide accurate information on all abortions that occur within their social networks. However, when applying these methods to measure stigmatized or hidden behaviors, this assumption is almost always violated, resulting in a phenomenon known as “transmission bias” (Salganik, Mello, et al. 2011; Bernard et al. 2010). The existence of abortion‐related stigma in most settings means that women will often have imperfect information about their peers’ abortions and thus cannot know of all the abortions that take place in their social networks. Without measuring the magnitude of and adjusting for this bias, the resulting abortion incidence estimates will be underestimated. For example, if respondents are only aware of 80 percent of the abortions that occur within their social networks, we assume the remaining 20 percent were not reported. To account for these missing abortions in this scenario, the corresponding abortion incidence estimate needs to be adjusted upwards by a factor of 1.25 (1/0.80) (Bernard et al. 2010; Salganik, Mello, et al. 2011). Researchers have argued that the magnitude of abortion‐specific transmission bias will vary based on how the method defines the social network. The assumption is that respondents will have better knowledge of abortions in their social networks when network definitions are limited to a small number of strong social ties, thereby reducing the magnitude of transmission bias (Bell et al. 2020; Giorgio, Sully, and Chiu 2021; Sedgh and Keogh 2019; Yeatman and Trinitapoli 2011). A recent review of these studies found that transmission bias was large even among these strong ties, with only 41 percent to 56 percent of women's close confidantes knowing about their abortions (Owolabi et al. 2023). Further, because the NSUM uses much broader definitions of social networks that encompass both strong and weak ties, it is likely that transmission bias rates will be even higher when this method is used to estimate abortion incidence.

As such, in order for the NSUM to generate accurate estimates of abortion incidence, it is essential to accurately document the visibility of abortions within broad social networks (i.e., networks that include both strong and weak social ties). The NSUM has been previously used to measure several stigmatized or hidden populations, such as men who have sex with men (MSM) and female sex workers (FSWs), and a recent literature review outlined different approaches that have been used to measure and adjust for transmission bias in these studies (Haghdoost et al. 2018). One common method, known as the social respect approach, uses respondent's self‐reported level of respect for members in the hidden population to adjust the number of individuals they report knowing in that hidden population (Guo et al. 2013; Rwanda Biomedical Center/Institute of HIV/AIDS, Disease Prevention and Control Department (RBC/IHDPC) et al. 2012; Sulaberidze et al. 2016; Wang et al. 2015). The theory behind this approach is that members of a stigmatized population will be more likely to disclose their membership status to respondents with higher levels of respect for that population. However, there are several problems with this approach. First, there is no empirical evidence to suggest the magnitude of the relationship between respect scores and transmission bias, or how this relationship may vary by other network factors. Additionally, Haghdoost et al. (2018) note that the way this estimate is calculated allows for an adjustment factor that is less than 1.0, meaning that this approach does not truly measure the concept of transmission bias and will sometimes reduce estimates of the number of individuals they know in the target population. As an alternative approach, a few studies have attempted to measure transmission bias through community‐based surveys. Individuals in the hidden population (identified through self‐reports) are asked for the number of people in their social network that they have disclosed their membership status to (i.e., how many people they told about their abortion), and that number is divided by the size of their social network (estimated through the NSUM). However, this approach has been shown to underestimate the visibility of men who have sex with men in Japan (Ezoe et al. 2012) as well as of women who have abortions in Uganda and Ethiopia (Sully, Giorgio, and Anjur‐Dietrich 2020), likely due to social desirability and selection biases in the self‐reported data.

A promising approach to estimate the social visibility of hidden behaviors or characteristics within broad social networks is the game of contacts method, developed by Salganik, Mello, et al. (2011). This method uses RDS to recruit members of the hidden population of interest and estimates the visibility of the hidden characteristic through a game‐like activity. Researchers have outlined several encouraging results from applications of the game of contacts among intravenous drug users; sensitivity tests did not suggest the existence of bias created by the design of the game, and there was little missing data (suggesting that respondents understood the game), disclosure patterns were consistent with previous theories, and the game produced visibility estimates that were plausible and/or consistent with external data (adding to face validity) (Maghsoudi et al. 2014; Salganik, Mello, et al. 2011; CIF 2015). That said, it is unknown whether the game of contacts methodology will be successful in measuring the visibility of abortions within broad social networks, as abortion represents a different type of behavior than those investigated in earlier studies. For example, while engaging in injection drug use or sex work often results in socially connected populations, obtaining an abortion does not necessarily involve social interaction with other women. If this is the case, women who have abortions may be less socially connected than other hidden populations (Rossier et al. 2021). At the same time, some researchers have argued that abortion may be more visible within social networks in certain types of social and legal environments, as individuals may seek out peers with abortion experiences to gain information about how to access abortion methods or providers (Rossier et al. 2021). One study conducted in Kerman, Iran, attempted to measure abortion visibility within broad social networks using a method somewhat similar to the game of contacts (although not in a game format) and found that the visibility of elective abortion was 8 percent among women's broad social networks (Zamanian et al. 2016). However, that study did not evaluate how well the method performed nor did it attempt to externally validate the resulting adjusted estimates of abortion incidence. As such, more work is needed to understand whether the game of contacts method can be used to measure the visibility of abortions.

This study uses data collected in Uganda and Ethiopia, which represent different legal contexts for abortion restrictions. In Uganda, women have limited access to safe abortion care as abortion is only legal to save a woman's life (Uganda Ministry of Health 2015). The law in Ethiopia is more liberal and allows for abortion under several legal grounds including when the woman's life or health is at risk; in cases of rape, incest, and fetal impairment; when the woman is a minor; and when the woman has physical or mental disabilities that leave her unprepared to bring up a child (Ethiopia Ministry of Health 2023). Despite these legal differences, several studies have shown that abortion‐related stigma is common in both Ethiopia and Uganda (Alemayehu et al. 2017; Atuyambe et al. 2015; Kebede, Middelthon, and Hilden 2018; Nyanzi, Nyanzi, and Bessie 2005). Further, a recent study found that women in both countries dramatically underreported their abortions when asked directly in surveys (Giorgio et al. 2023), a phenomenon that is largely attributable to abortion‐related stigma (R. K. Jones and Kost 2007; Lindberg et al. 2020).

There is some existing evidence on the incidence of induced abortion in Uganda and Ethiopia. The most recent studies that produced abortion incidence estimates in both countries were conducted roughly a decade ago, estimating abortion incidence at 39 abortions per 1000 women aged 15–49 in Uganda (2013) and 28 per 1000 in Ethiopia (2014) (Moore et al. 2016; Prada et al. 2016). Those studies also revealed that abortion rates were the highest among adolescents in both countries after adjusting for recent sexual activity (76.1 per 1000 in Uganda and 90.7 per 1000 in Ethiopia) (Sully, Atuyambe, et al. 2018; Sully, Dibaba, et al. 2018). That said, it is possible that abortion incidence rates have changed in the preceding 10 years. A more recent study that used a global Bayesian model estimated annual abortion incidence for 2015–2019 at 43 abortions per 1000 women aged 15–49 in Uganda and 24 per 1000 in Ethiopia (Bearak et al. 2022). However, because the model relies heavily on previous abortion incidence studies to predict its country‐specific estimates, the stability of these estimates over time is likely a function of a lack of new data on abortion incidence (Bearak et al. 2022). The most recent attempt to measure abortion incidence using primary data collection involved an NSUM module added to the 2018 round of the Performance Monitoring for Action (PMA) surveys in both countries (Sully, Giorgio, and Anjur‐Dietrich 2020). That application of the NSUM was shown to produce sufficiently generalizable samples from respondent's social networks (Sully, Giorgio, and Anjur‐Dietrich 2020). However, as noted above, the approach that was employed to estimate transmission bias failed and, as a result, that work ultimately did not generate updated abortion incidence rates.

This paper shares the results from an exploratory study of the performance of the game of contacts to estimate abortion visibility within two sites in Uganda and Ethiopia. Results from this work will help determine whether it could be used in conjunction with future applications of the NSUM to generate nationally representative estimates of abortion incidence. First, we use descriptive analyses and sensitivity tests recommended by Salganik, Mello, et al. (2011) to evaluate how well the game of contacts methodology performed among the study samples in each setting. Next, we compare each country's abortion visibility estimates by key abortion‐related indicators to provide insight into the performance of the game of contacts in measuring abortion visibility. While the goal of this study is not to produce updated abortion incidence estimates, we do conduct a crude external validity test by using our estimates of abortion visibility to adjust the 2018 NSUM‐derived abortion incidence estimates in Uganda and Ethiopia, as presented by Sully, Giorgio, and Anjur‐Dietrich (2020). Given that this application of the game of contacts was not nationally representative in either country, this comparison is less than ideal. However, the level of comparability between this study's newly adjusted NSUM abortion incidence rates and what is currently known about abortion incidence in each country will help determine whether the game of contacts estimates of abortion visibility are plausible versus potentially unreasonable. While our analyses indicate that the mechanics of the game of contacts performed well in each setting, we also identify concerns about the validity of the study's visibility estimates due to issues with the RDS‐generated study samples and differences in respondent's understanding of key questions in the NSUM and game of contacts modules. We conclude by making recommendations for how applications of this methodology could be improved in the future to better estimate and adjust for abortion‐related transmission bias in social network–based estimates of abortion incidence.

## METHODS

### Sampling and Recruitment

Data for this analysis were collected from May to August 2021 in Uganda and April to May 2021 in Ethiopia.^1^ In each country, we recruited two samples of women of reproductive age who had an abortion: one in an urban setting (Kampala and Addis Ababa) and one in a rural/peri‐urban setting (Rakai and rural Oromia). To be eligible for participation, women must have been between the ages of 15 and 49, have had an abortion within the last six years, and lived in one of the recruitment catchment areas.

This study uses RDS to sample and recruit women who have had an abortion. RDS uses a chain referral technique designed to reach deep into social networks, providing access to hidden populations for which no sampling frame exists (Heckathorn 1997). A detailed description of the RDS process used in this study is described elsewhere (Giorgio et al. 2022). In brief, study staff worked with health facilities and community outreach groups that serve women who have abortions to identify the 40 initial seed participants (Addis Ababa *n* = 8, Oromia *n* = 16, Kampala *n* = 11, Rakai *n* = 5). After participating in the study survey (described below), the seeds were offered up to three coupons with which to recruit peers who also met the study eligibility criteria. Successfully recruited peers who traveled to one of the study sites and participated in the in‐person quantitative interview became the first wave of RDS participants. After participation, these individuals became recruiters and were also given up to three coupons with which to recruit their peers. In a traditional RDS, this process would continue through multiple waves of recruitment until equilibrium on key variables is reached. Given the exploratory and formative nature of this current work, recruitment stopped once a predetermined sample size based on budgetary restrictions was reached (*n* ∼ 400 in Uganda and *n* ∼ 250 in Ethiopia).

Recruitment lasted between 6.5 and 7.5 weeks, and final sample sizes were 416 in Uganda and 254 in Ethiopia. Overall, the RDS methodology performed relatively well in each site; equilibrium was reached on most key variables, homophily indices did not indicate a clear preference for in‐ or out‐group recruitment, and few violations to the key RDS assumptions were identified (Giorgio et al. 2022). That said, some evidence suggests that the final samples may not have represented the true underlying population of women who have had an abortion in each setting. First, the sociodemographic characteristics of women in the RDS samples differed from those in the most recent Demographic and Health Survey samples of reproductive‐age women living in the four study areas. However, the observed differences in age, education, and marital status are consistent with known differences between all women of reproductive age and those who have had an abortion in many settings. Thus, it is difficult to determine whether these differences represent biases in the RDS samples or the true characteristics of women who have had an abortion in the study areas. In addition, the study's reliance on health workers to identify initial seeds likely led to an overrepresentation of women who had facility‐based abortions, particularly in the Ugandan samples. Finally, an important feature of a successful RDS is ensuring there is sufficient mixing of members of the target population across the recruitment chain. In this study, it became clear that one highly productive recruitment chain in Kampala had tapped into networks of FSWs. This raised concerns that the sample in this chain would have specific sociodemographic characteristics compared to the others, introducing bias into the sample and the resulting estimates. Further, the speed at which these mainly FSW‐populated chains progressed compared with other chains meant that, if we had not intervened, FSWs would be overrepresented in our final sample. While abortions are likely common among FSWs, these women represent a very small proportion of all women aged 15–49 in both countries, and their abortion experiences are not necessarily representative of those of the general population. As such, study staff decided to purposely end these chains once this issue was discovered. However, it is possible that the experiences of FSWs are still overrepresented in the final samples.

The Institutional Review Boards of the Guttmacher Institute, Johns Hopkins Bloomberg School of Public Health, Makerere University, and Addis Ababa University as well as the Uganda National Council for Science and Technology provided ethical approval for this study.

### Survey Instrument

All respondents participated in a face‐to‐face quantitative survey administered by trained study staff, and responses were recorded using Open Data Kit (ODK) software on Android smartphones. The quantitative survey collected information on sociodemographic characteristics, including age, marital status, educational status, and whether the respondent worked for money in the last three months. The survey also gathered information about the respondent's most recent abortion, including timing (more or less than two years ago), location (in or out of a health facility), gestational age, and whether the respondent experienced any abortion‐related health complications.

The survey also included the game of contacts module. The game of contacts is designed to estimate abortion visibility within social networks by eliciting information on people within the respondent's social network. In this study, we used the same broad social network definition as was used to generate the previous NSUM abortion incidence estimates in Uganda and Ethiopia; network members (which we refer to as “alters”) are women, the respondent knows by sight and name, who live in the same country as her, who are between the ages of 15 and 49, and with whom the respondent has had contact in the last year (Sully, Giorgio, and Anjur‐Dietrich 2020). To generate a random sample of each respondent's alters, interviewers used a deck of 24 cards of first names. As with previous applications of the game of contacts, these first names included rare, somewhat common, and more common first names of women (Salganik, Mello, et al. 2011). We used four different lists of names specific to each study site to reflect the frequency of names within the particular context (Online Appendix A). The deck of cards is shuffled before each interview to ensure that names appear in random order for each respondent.

**FIGURE 1:**
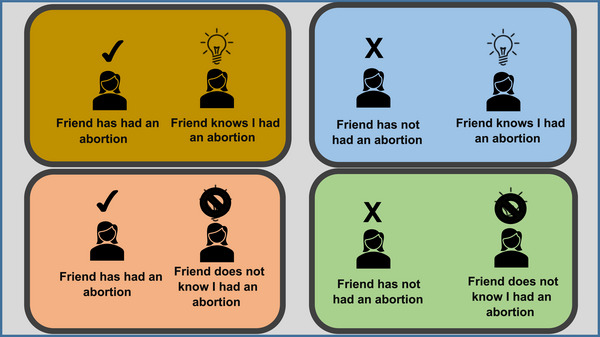
Game of contacts board used for identifying social network members by their abortion status and whether they knew the respondent had an abortion

The game of contacts is played as follows:

1. The interviewer shuffles a deck of 24 playing cards containing first names.
2. The interviewer flips over the card on the top of the deck to reveal the name (e.g., “Mirembe”).
3. The respondent is asked how many women she knows named Mirembe who fit the study's social network definition.
4. The respondent picks up a token for each woman she knows with that name.
5. The respondent is instructed to place the token onto a 2×2 board, such that the location of each token indicates whether the “Mirembe” she's referring to has ever had an abortion and whether that Mirembe knows if the respondent herself has ever had an abortion (Figure 1). If the respondent knows more than one person named Mirembe who meets the social network definition, this process of placing tokens is repeated for each “Mirembe.”
6. Once the respondent has provided information on all “Mirembes” she knows, the interviewer records how many “Mirembe” tokens are on each location and clears the board.
7. The interviewer flips over the next card from the deck.  This process is continued until the respondent has reported on all 24 names.

Information from the game of contacts is used to calculate the proportion of respondents’ alters who know whether the respondent has had an abortion and the proportion of alters whose abortion the respondent knows about.

### Analysis Plan

First, we describe the results of the RDS sampling and recruitment process in each of the four sites, including the number of initial seeds, recruitment chain lengths, the average number of women in each recruitment chain, and the total sample sizes. We provide descriptive statistics for sociodemographic and abortion‐related characteristics for each sample. We also test for differences in these characteristics by study site within each country using chi‐squared tests for significance.

### Game of Contacts Performance and Sensitivity Analyses

We present several descriptive indicators of game performance in each of the four sites, including the distribution of where alters tokens were placed on the game board, the average number of alters identified, the average number of alters who had had an abortion, the proportion of respondents who reported knowing zero alters, and the proportion of respondents who said they did not know where to place at least one of their identified alters on the game board. To further investigate the performance of the game of contacts in this study, we employed two sensitivity tests recommended by Salganik, Mello, et al. (2011):

- *Question order effects*: There is the possibility that respondents could grow tired of playing the game, leading them to underreport the number of known alters and alters’ abortion knowledge as the game progressed through the 24 cards. To investigate whether such “game fatigue” occurred in our study, we calculated the average number of known alters and the proportion of alters that knew of the respondent's abortion for each round of the game. Because the game is played with a physical deck of cards that the interviewer shuffles at the start of every interview, each name is asked in a random order for every respondent. As such, the average number of reported alters and the proportion of alters that are aware of the respondent's abortion should remain relatively constant throughout the game if fatigue was not an issue.
- *Name effects*: We investigated whether our selection of names may have biased our estimates of abortion visibility. First, we calculated the average number of identified alters with each name and the proportion of alters with each name who knew about the respondent's abortion. Because we selected names that were common, somewhat common, and rare in each setting, we expect that the frequency of identified alters with each name should vary. However, the assumptions underlying the game of contacts stipulate that knowledge of the respondent's abortion should be independent of the alters’ first name, so evidence to the contrary could indicate bias introduced by inappropriately chosen names. We also assessed variation in visibility estimates after iteratively dropping one name at a time. Relatively stable visibility estimates throughout this process indicate that the inclusion of any individual name is not unduly biasing the results.

### Estimating Abortion Visibility and Transmission Bias

To calculate abortion visibility in each setting, we use the following formula, as described by Salganik, Mello, et al. (2011):

$$\tau =\frac{\sum_{i\in T}\sum_{j=1}^{d_{i}}I\left(a_{\mathrm{ij}}\right)}{\sum_{i\in T}d_{i}}$$

where τ is the average visibility of abortion respondents’ social networks, *d_i_* is the personal network size of person *i, a_i,_*_1_ − *a_i,di_* is the alters of person *i*, and *T* is the set of people who have had an abortion in the underlying population. In this equation, function *I* is equal to 1 if alter *j* knows that respondent *i* had an abortion and 0 otherwise. Possible values of *τ* range from 0 (meaning that abortion is completely invisible in the underlying population) to 1 (meaning that everyone has perfect knowledge of abortions within their networks). After calculating the overall estimate of abortion visibility within each site, we additionally investigate whether abortion visibility differs by abortion characteristics.

To estimate a single transmission‐bias factor estimate for each country, we combined the site‐specific estimates of abortion visibility. In each country, our two sites represented one urban and one rural setting. While there is no reliable information on urban/rural differences in the rate of abortion in Uganda or Ethiopia, we hypothesized that abortion visibility could vary geographically. As such, we do not average the site‐specific estimates together. Instead, we weight them by the most recent World Bank estimates for the urban/nonurban distribution of the underlying population of women of reproductive age in Uganda and Ethiopia.^2^

### Estimating Abortion Incidence Rates in Uganda and Ethiopia

We first present the induced abortion incidence rates for Uganda and Ethiopia from Sully, Giorgio, and Anjur‐Dietrich (2020) that were estimated using the NSUM and not adjusted for transmission bias (Sully, Giorgio, and Anjur‐Dietrich 2020). We then take the inverse of the combined estimates of abortion visibility generated through our application of the game of contacts and apply them to the corresponding unadjusted rates to generate a transmission bias–adjusted abortion incidence estimates for Uganda and Ethiopia (Salganik, Mello, et al. 2011). For example, if our study resulted in a $\hat{\tau }$= 0.4 (meaning that, on average, individuals are aware of 40 percent of all abortions that occurred to members of their broad social networks), we would inflate our corresponding incidence estimate by a factor of 2.5 (1/0.40).

Despite efforts to design the game of contacts module to produce a measure of transmission bias that would be directly applied to these previous NSUM incidence estimates, the research team discovered that women in each study were likely using different definitions of what it means to “know” if someone has had an abortion. In the game of contacts, respondents were asked to report whether each alter knew about the respondent's abortion. Reports from the field indicate that most respondents understood this question to mean that the respondent told the alter directly about her abortion. In addition, in many cases, respondents would not know whether an alter learned about the respondent's abortion indirectly (through another person or source) and therefore would not be able to report this knowledge in the game. However, the published NSUM incidence estimates for Uganda and Ethiopia were based on abortions that respondents learned through two pathways: (1) directly from the woman who had the abortion and (2) indirectly through gossip or other sources.

To account for this difference, we also estimate a third abortion incidence rate that adjusts for direct versus indirect transmission of abortion information. First, we recalculated the Ethiopia NSUM abortion incidence rate to only include abortions that respondents were told about directly. Similar information is not available from the Uganda NSUM module. Instead, we assumed that a similar proportion of women in Uganda and Ethiopia reported on abortions that they knew about indirectly, and thus we reduced the unadjusted estimate from Uganda accordingly. We then inflate these estimates by the transmission bias factor described above.

Analyses were performed using Stata version 16.0.

## RESULTS

### Descriptive Statistics and Game of Contacts Performance

The number of initial RDS seeds ranged from 5 in Rakai to 16 in rural Oromia (Table 1). The Rakai site resulted in the largest average number of recruitment waves and recruitment chain length (8.1, 20, respectively), and Oromia was the smallest (3.4, 8). The average number of recruitment waves and longest chain lengths were similar in the two urban sites (Kampala = 4.8, 10; Addis Ababa = 5.0, 10). The combined total sample sizes were 416 in Uganda and 254 in Ethiopia.

**TABLE 1:** Sampling and recruitment for each study site in Ethiopia and Uganda

	Ethiopia		Uganda	
	Addis Ababa	Oromia	Kampala	Rakai
Total number of seeds	8	16	11	5
Average number of waves of recruitment	5.0	3.4	4.8	8.1
Longest wave	10	8	10	20
Total sample size	86	168	119	297

In Uganda, 38.0 percent (*n* = 158) of the sample were aged 30 or older, and adolescents (15–19) represented the smallest proportion of respondents (6.3 percent, *n* = 26) (Table 2). Approximately one‐third of women (34.6 percent, *n* = 144) were currently married or living with a partner, and 42.8 percent (*n* = 178) were formerly married. All respondents had at least some formal schooling, and 52.4 percent (*n* = 218) had an educational attainment of secondary or higher. Most women (80.3 percent, *n* = 334) had worked for wages in the past three months. Approximately one‐quarter (23.8 percent, *n* = 99) reported having multiple lifetime abortions. For respondents’ most recent abortions, the majority occurred in the last two years (60.1 percent, *n* = 250), were performed in a health facility (74.7 percent, *n* = 310), and resulted in a health complication (as reported by the respondent) (73.1 percent, *n* = 304). 16.3 percent of respondents (*n* = 68) reported that their most recent abortion occurred during the second trimester of pregnancy. Women's sociodemographic characteristics were similar in Ugandan sites (urban and rural/peri‐urban), although a larger proportion in Rakai than in Kampala had worked for wages in the past three months (85.9 percent vs. 66.4 percent). More differences by site were observed for abortion characteristics; a larger proportion of respondents in Kampala than in Rakai reported multiple lifetime abortions (34.5 percent vs. 19.5 percent), and women in Rakai were more likely than those in Kampala to report a second‐trimester abortion (20.2 percent vs. 6.7 percent) or a postabortion complication (75.8 percent vs. 66.4 percent).

**TABLE 2:** Sociodemographic and abortion characteristics overall, and by recruitment site in Uganda and Ethiopia^a^

	Uganda						Ethiopia					
	Overall		Kampala		Rakai		Overall		Addis Ababa		Oromia	
	(*N* = 416)		(*N* = 119)		(*N* = 297)		(*N* = 254)		(*N* = 86)		(*N* = 168)	
	*n*	%	*n*	%	*n*	%	*n*	%	*n*	%	*n*	%
Sociodemographic characteristics												
Age												
15–19	26	6.3	9	7.6	17	5.7	19	7.5	**3**	**3.5**	**16**	**3.5**
20–24	123	29.6	33	27.7	90	30.3	69	27.2	**12**	**14.0**	**57**	**33.9**
25–29	109	26.2	30	25.2	79	26.6	64	25.2	**23**	**26.7**	**41**	**24.4**
30–49^b^	158	38.0	47	39.5	111	37.4	102	40.2	**48**	**55.8**	**54**	**32.1**
Current marital status												
Married/living with partner	144	34.6	46	38.7	98	33.0	71	28.0	**37**	**43.0**	**34**	**20.2**
Never married	94	22.6	32	26.9	62	20.9	89	35.0	**19**	**22.1**	**70**	**41.7**
Formerly married (divorced/widowed)	178	42.8	41	34.5	137	46.1	94	37.0	**30**	**34.9**	**64**	**38.1**
Highest level of education												
None	0	0.0	0	0.0	0	0.0	37	14.6	13	15.1	24	14.3
Primary	198	47.6	50	42.0	148	49.8	128	50.4	46	53.5	82	48.8
Secondary or higher	218	52.4	69	58.0	149	50.2	89	35.0	27	31.4	62	36.9
Worked for money in the past three months	334	80.3	**79**	**66.4**	**255**	**85.9**	185	72.8	63	73.3	122	72.6
Abortion characteristics												
Multiple lifetime abortions	99	23.8	**41**	**34.5**	**58**	**19.5**	61	24.0	18	20.9	43	25.6
Most recent abortion characteristics												
< two years ago	250	60.1	73	61.3	177	59.6	129	50.8	38	44.2	91	54.2
Second trimester	68	16.3	**8**	**6.7**	**60**	**20.2**	20	7.9	4	4.7	16	9.5
Facility‐based abortion	310	74.7	91	77.1	219	73.7	210	82.7	**59**	**68.6**	**151**	**89.9**
Postabortion complication	304	73.1	**79**	**66.4**	**225**	**75.8**	93	36.6	**42**	**48.8**	**51**	**30.4**

^a^ Bold indicates statistically significant differences by region.

^b^ Women aged 30–49 were grouped into one category as opposed to five‐year age groupings due to the small sample size of women aged 35—49.

The combined sample in Ethiopia showed similar distributions of age, marital status, and income‐earning work in the last three months compared with Uganda (Table 2). However, Ethiopian respondents had lower levels of education, with 14.6 percent (*n* = 37) reporting no formal schooling, and only 35.0 percent (*n* = 89) with an education level of secondary or higher. Like the Ugandan respondents, approximately one‐quarter of those in Ethiopia (24.0 percent, *n* = 61) reported multiple lifetime abortions. Approximately half (50.8 percent, *n* = 129) had their most recent abortion within the past two years, and most accessed it from a health facility (82.7 percent, *n* = 210). Second‐trimester abortions and health complications were far less common in Ethiopia than in Uganda (7.9 percent, *n* = 20 and 36.6 percent, *n* = 93, respectively). There were also key differences between study sites in Ethiopia. Larger proportions of women in Addis Ababa than in rural Oromia were in the oldest age group (30 + : 55.8 percent vs. 32.1 percent) or currently married/living with a partner (43.0 percent vs. 20.2 percent). Additionally, facility‐based abortions were less common in Addis Ababa than in rural Oromia (68.6 percent vs. 89.9 percent), whereas postabortion complications were more common in Addis Ababa compared with rural Oromia (48.8 percent vs. 30.4 percent).

The game of contacts resulted in the identification of 6144 alters in Uganda and 3712 in Ethiopia (Table 3). In both countries, most respondents reported that identified alters were unaware of the respondent's abortion and the alter had not had an abortion herself (Uganda = 4174, Ethiopia = 2852). On average, respondents identified approximately 15 alters through the game, with similar averages in both countries (Uganda: 14.8, *SD* = 16.0; Ethiopia: 14.6, *SD* = 10.4) (Table 4). The average number of alters who were identified as having had an abortion by each respondent was slightly higher in Uganda (3.8, *SD* = 5.4) than in Ethiopia (2.2, *SD* = 3.8). Across all sites, a small percentage of respondents reported having zero alters (Kampala = 2.5 percent, Rakai = 2.7 percent, Addis Ababa = 0 percent, Oromia = 1.2 percent), and an even smaller proportion had difficulty placing one of their identified alters in the appropriate square on the board (Kampala = 0.8 percent, Rakai = 1.0 percent, Addis Ababa = 1.2 percent, Oromia = 0 percent).

**TABLE 3:** Game of contacts results for Uganda and Ethiopia

	Uganda		Ethiopia	
	(*n* = 6144)		(*n* = 3712)	
	Alter had an abortion	Alter has not had an abortion	Alter had an abortion	Alter has not had an abortion
Alter knew of respondent's abortion	835	398	294	286
Alter did not know about the respondent abortion	725	4174	277	2852

**TABLE 4:** Descriptive statistics for the game of contacts results for Uganda and Ethiopia

	Uganda			Ethiopia		
	Overall	Kampala	Rakai	Overall	Addis Ababa	Oromia
	(*n* = 416)	(*n* = 119)	(*n* = 297)	(*n* = 254)	(*n* = 86)	(*n* = 168)
Average number of alters identified, mean (*SD*)	14.8 (16.0)	14.4 (13.1)	14.9 (17.0)	14.6 (10.4)	16.6 (10.9)	13.6 (10.1)
Average number of abortions identified among alters, mean (*SD*)	3.8 (5.4)	6.4 (8.4)	2.7 (3.0)	2.2 (3.8)	2.0 (3.0)	2.4 (4.2)
Proportion of respondents who reported 0 alters, *n* (%)	11 (2.6%)	3 (2.5%)	8 (2.7%)	2 (0.8%)	0 (0%)	2 (1.2%)
Proportion of respondents who could not place at least 1 identified alter on the board, *n* (%)	4 (1.0%)	1 (0.8%)	3 (1.0%)	1 (0.4%)	1 (1.2%)	0 (0%)

The results of our sensitivity analyses did not reveal the existence of either question order or name effects, suggesting that the results were not unduly biased by either of these factors. (See Online Appendix B for results and detailed explanation.)

### Abortion Visibility and Updated Incidence Estimates

**FIGURE 2:**
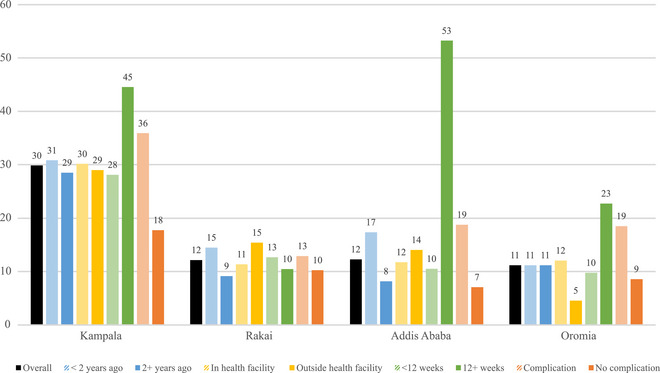
Abortion visibility estimates, overall, and by abortion characteristics in each site

Overall, abortions were most visible in Kampala, with an estimated 30 percent of respondent's social network members being aware of their abortion (Figure 2). Lower levels of abortion visibility were observed in Rakai (12 percent), Addis Ababa (12 percent), and rural Oromia (11 percent). We observed differences in abortion visibility by abortion characteristics within sites, and some patterns in these results across sites emerged. Most consistently, abortions that resulted in health complications were more visible than those that did not, and second‐trimester abortions were the most visible in all sites, except Rakai. In Rakai and Addis Ababa, abortions that occurred in the past two years were more visible than less recent abortions (Rakai: 15 percent vs. 9 percent, Addis Ababa: 17 percent vs. 8 percent), and differences by recentness were small or nonexistent in Kampala and rural Oromia. No consistent pattern in abortion visibility emerged in whether the abortion occurred within or outside a health facility.

**FIGURE 3:**
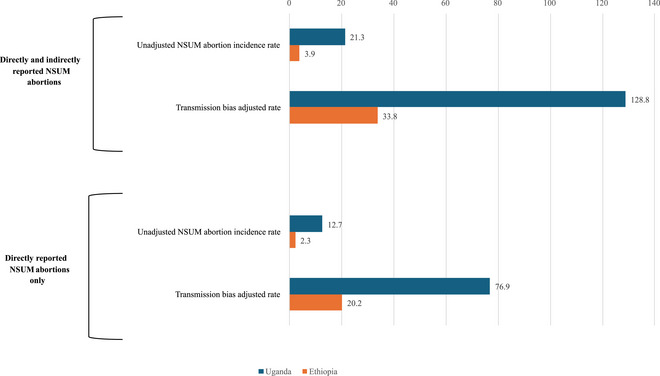
One‐year induced abortion incidence rates per 1000 women aged 15–49 for Uganda and Ethiopia

After accounting for the urban/nonurban population distribution in each country, the combined estimates of abortion visibility were 16.6 percent in Uganda and 11.4 percent in Ethiopia, corresponding to transmission bias factors of 6.02 and 8.77, respectively. Figure 3 displays the results of applying these transmission bias estimates to abortion incidence rates for each country. Before adjusting for transmission bias, the NSUM incidence rates presented by Sully et al. (2020) were 21.3 per 1000 women of reproductive age in Uganda and 3.9 per 1000 in Ethiopia. After adjusting for transmission bias, the one‐year abortion incidence rates increase to 128.8 per 1000 women in Uganda and 33.8 per 1000 in Ethiopia. However, these are likely overestimates given the inclusion of both direct and indirect knowledge of abortion in the NSUM incidence estimates. In Ethiopia, approximately 57 percent of women who reported an abortion in the NSUM module learned of the event directly from the person who had the abortion. After recalculating the NSUM estimates to only account for directly reported abortions, the unadjusted rates decrease to 12.7 per 1000 women in Uganda and 2.3 per 1000 women in Ethiopia. As such, our final one‐year abortion incidence estimates, adjusted for direct reporting and transmission bias, were 76.9 per 1000 women in Uganda and 20.2 per 1000 women in Ethiopia.

## DISCUSSION

The results from this exploratory study provide important insights into whether the game of contacts methodology can be used to estimate the visibility of abortions within women's broad social networks. While we identified some promising aspects of the game's performance, we also identified concerns with the implementation of the RDS and game of contacts methodology that may have produced biases in this study's estimates of abortion visibility and abortion incidence. We thus discuss several areas where the game of contacts methodology can be adjusted and improved in future studies aimed at assessing abortion visibility in restrictive settings.

Our descriptive results, as well as the results of our sensitivity tests, did not identify any concerns with the performance of the “game” itself in both countries. Respondents were able to identify a substantial number of alters through the game, and few had difficulty placing each alter on the appropriate square on the game board (i.e., the square corresponding to the alter's knowledge of the respondent's abortion status and vice versa). Our sensitivity analyses did not reveal question‐order or name‐selection effects. There is also some evidence to suggest that the playing the game of contacts helped respondents in identifying more members of their social networks who were aware of their abortion. While previous attempts to measure abortion visibility from representative surveys resulted in low estimates (5.3 percent of women's broad social networks in Uganda and 2.3 percent in Ethiopia) (Sully, Giorgio, and Anjur‐Dietrich 2020), this study's visibility estimates using similar social network definitions are higher: approximately 16.6 percent in Uganda and 11.4 percent in Ethiopia. As such, it is likely, that the game of contacts method presents an improvement in measuring abortion visibility as compared to previous attempts to measure it directly from women who self‐reported abortions in representative surveys. That said, it is likely that the existence of other biases impacted the validity of this study's estimates of abortion visibility.

### Biases in Transmission Bias Estimates

Our results suggest lower levels of abortion visibility in social networks in Ethiopia as compared to Uganda. This is consistent with previous work, which suggests that women may be less likely to disclose their abortions in contexts where abortion is more legally accessible since they do not need to learn from others where or how to gain access to abortion services (Rossier et al. 2021). Looking at visibility patterns by abortion characteristics, we consistently observed that abortions that resulted in health complications and those that occurred in the second trimester were more visible than those that did not. It may be that these types of abortions are more likely to be revealed through extenuating circumstances as opposed to women choosing to disclose this information to their friends or relatives. For example, social network members may have already known about the respondent's pregnancy prior to a second‐trimester abortion or may have needed to assist the respondent while she was recovering from a postabortion complication.

While it is encouraging that the differences we observed in transmission bias estimates across contexts and abortion characteristics are consistent with prior work, this consistency is not sufficient to conclude that we accurately estimated the visibility of induced abortions in this study. One potential contributor to biases in our visibility estimates is whether our RDS samples were indeed representative of all women who have abortions in each study site. Our reliance on identifying initial seeds from health care workers/health facility settings likely resulted in an overrepresentation of facility‐based abortions (Giorgio et al. 2022). Previous research has shown that seeds do not need to be representative of the underlying population, but they should represent the different types of ties within a social network (Salganik and Heckathorn 2004; Wejnert and Heckathorn 2008). If women who induce abortions outside of formal health facilities are more likely to be connected to one another because they share information about how to access these informal resources, our approach to seed selection may have resulted in an overrepresentation of facility‐based abortions. The most recent estimate of the proportion of abortions occurring in health facilities in Ethiopia was 53 percent in 2014 (Moore et al. 2016). While this proportion has likely increased in the intervening years, it may not have increased to the level documented in this study (83 percent), especially given the increasing access to non‐facility‐based medication abortion during the same time frame. While no similar baseline estimate exists for the proportion of facility‐based abortions in Uganda, it is highly unlikely that the 75 percent reported in this study is accurate given the highly restrictive nature of abortion in the country. That said, it is unclear how problematic the issue of overrepresenting facility‐based abortions is for our estimates of abortion visibility. In the two urban sites, there was virtually no difference in transmission bias estimates based on whether a woman's abortion occurred within a facility. We did observe differences in the rural sites but in opposite directions. (In Rakai facility‐based abortions were slightly less visible, while they were much more visible in Oromia). As such, it is unclear the extent to which the overrepresentation of facility‐based abortions in our sample biased our transmission estimates or the direction of any resulting bias.

A second (and potentially more problematic) concern is that our application of RDS might have oversampled specific subgroups of women. As previously noted, we discovered during fieldwork that one recruitment chain in Kampala was progressing faster than others, and study staff suspected that this chain was tapping into a well‐connected network of FSWs. In response, a decision was made to intentionally end this recruitment chain. However, it is possible that the final Kampala sample overrepresents the experiences of FSWs. When we compare abortion visibility by study site, we find that the higher visibility of abortions in Uganda is largely being driven by respondents in Kampala, with results from Rakai being comparable to both sites in Ethiopia. It is possible that well‐connected networks of FSWs may be more likely to share abortion information with one another compared to women in the general population, thus explaining the increased visibility of abortions that we observed in Kampala.

The final main concern about the representativeness of our RDS samples is not unique to this study and instead highlights the underlying question of the appropriateness of using the game of contacts to measure abortion visibility. Because RDS relies on social connections and social networks for sampling and recruitment, the most isolated or socially disconnected members of a network are likely to be underrepresented in the data. Further, because abortion is not an inherently networked behavior, it is possible for a woman to have an abortion without anyone else in her social network knowing, meaning that she has a 0 percent probability of being sampled through RDS. Both phenomena would inappropriately inflate transmission bias estimates, making it appear that abortions are more visible than they really are. This limitation is specific to measuring abortion visibility as compared to the visibility of other behaviors such as injecting drugs or sex work FSWs, as these behaviors often require social connections to access necessary materials or spaces and occur consistently over a period of time (Salganik, Mello, et al. 2011; Maghsoudi et al. 2014). Conversely, while abortion is common, it is a time‐limited event that can occur without any interaction with one's social network. We are unable to assess the impact that nonnetworked abortions would have on the visibility estimates in our study, and no evidence exists on the proportion of all abortions that could be nonnetworked in our study populations. Not accounting for the missing, nonnetworked abortions would result in an overestimate of abortion visibility using the game of contacts.

### Biases in Abortion Incidence Estimates

There is utility to understanding how visible abortions are within social networks in and of itself. Such information can be used to better describe and contextualize levels of abortion stigma and provide insight into circumstances under which abortions occur. That said, researchers are primarily interested in measuring abortion visibility to adjust abortion incidence estimated using social network–based methods for transmission bias. One of the most difficult challenges in the field of abortion measurement is that there is often no gold standard with which to externally validate new estimates of abortion incidence. In many countries, there is simply no available data, or what does exist is of poor quality or is too dated to be useful. However, recent global modeling work that uses available historical and current data on induced abortion, contraceptive use, and the number and planning status of births has generated global, regional, and country‐specific estimates of abortion incidence over time (Bearak et al. 2022). That work's standardized modeling method and strict protocol for what types of data are included make these country‐specific estimates helpful yardsticks with which to compare new estimates of abortion incidence (Bearak et al. 2019). According to that model, abortion incidence for 2015–2019 in Ethiopia is 24 abortions per 1000 women (95 percent UI 17, 35), which is similar to this study's adjusted estimate of 20.2 per 1000 women (Bearak et al. 2022). However, our adjusted rate of 76.9 per 1000 for Uganda is much higher than (and outside of the uncertainty interval of) the modeled rate of 43 per 1000 women (95 percent UI 29, 60) (Bearak et al. 2022), suggesting that our study may have incorrectly estimated transmission bias.

It is unlikely that the two sources of bias outlined above (overrepresentation of FSWs and influence of nonnetworked abortions) explain the difference between the modeled abortion incidence estimate and this study's estimate in Uganda. Both of those sources of bias would make abortions appear more visible within social networks than they truly are, which would lead to an *underestimation* of abortion incidence. Given the fact that our study's Ugandan incidence estimate is almost double that of the modeled one, any influence these phenomena have on our abortion visibility estimates does not explain this large discrepancy.

One plausible explanation for the differing results is that our study likely inadequately accounted for differences in direct versus indirect transmission of information about abortions in one's social network. As noted above, reports from the field indicate that respondents interpreted the “game” question of whether an alter knew of their abortion to mean that the respondent directly disclosed this information to the alter. Of course, it is impossible for respondents to report cases of indirect abortion knowledge transfer if they are unaware of its occurrence. Thus, our study's estimates of abortion visibility should be interpreted as the visibility of abortions that respondents directly disclosed to members of their social networks. However, we know from previous work that women learn of abortions both directly (from the person who had the abortion) and indirectly (through gossip or other channels) (Sully and Giorgio 2021; Sully, Giorgio, and Anjur‐Dietrich 2020; Rossier et al. 2021). The 2018 NSUM module in Ethiopia included questions about how women knew of the abortions they were reporting, and 43 percent indicated that they had obtained this information *indirectly*, meaning they were not told about the abortion directly from the network member. Unfortunately, similar questions were not available in the Uganda NSUM module. To account for differences in the interpretation of “knowing” of someone's abortion between the NSUM and game of contacts, we recalculated the unadjusted NSUM abortion incidence rates to only include the abortions that respondents indicated they learned of directly. Due to data availability limitations in Uganda, we were forced to assume that similar proportions of women in Uganda and Ethiopia learned of abortions directly. However, learning about abortion second hand might be more common in Uganda than in Ethiopia. Some research supports this theory; women in Ethiopia described their cultural environment as one that discourages women from discussing issues around sexual activity and sexuality (Kebede, Hilden, and Middelthon 2014). If indirect knowledge transmission about abortion is indeed more common in Uganda than in Ethiopia, then our methodology would have inappropriately inflated the Ugandan abortion incidence estimate.

Moreover, our country‐specific estimates of transmission bias do not, in fact, represent the true underlying visibility of abortion in each country Our estimates of abortion visibility came from two small study sites in an urban and rural/peri‐urban location in each country, and the experiences of women who live in those areas may not represent abortion visibility for all women living in either country. Future work should investigate the extent to which abortion visibility varies by regional or geographic differences. It is also important to note that our study only adjusts for one form of potential bias in the NSUM estimates. Feehan and Salganik (2016) have identified two other potential sources of biases in the NSUM estimates that we have not adjusted for—barrier bias through nonrandom mixing and social desirability bias. If either of these other sources of potential bias are large enough, they could further explain the particularly high estimate of abortion incidence in Uganda and would suggest the need for adjusting for further sources of bias (Feehan and Salganik 2016).

## CONCLUSIONS AND RECOMMENDATIONS

While the abortion visibility estimates produced by this exploratory study were likely subject to bias, our analysis suggests that the game of contacts holds promise for measuring abortion‐related transmission bias. However, before that can be successfully achieved, several aspects of the design and implementation of this method should be modified and improved upon. First, future work should adhere more strictly to RDS implementation protocols as well as incorporate recommendations laid out by the recent RDS abortion studies (Giorgio et al. 2022; Zan et al. 2024). This will likely reduce some of the biases in RDS‐generated samples of women who have abortions that were present in this study. Next, it is essential that future social network–based research on abortion carefully define and account for both direct and indirect sharing of abortion information within social networks. This applies to the questions included in surveys to estimate incidence as well as to the questions in the game of contact module. Ensuring that both types of information sharing are clearly defined and captured and are comparable between the RDS and the game of contacts, will reduce many of the limitations of this current study. Finally, research is needed to investigate whether abortion visibility differs based on how abortion information is shared within social networks as well as by geographic areas within the country. The results of such future work will inform how to best produce nationally representative estimates of abortion incidence using social network–based methods. That research will also enable a better understanding of the role that social connections and social support play in how women obtain and experience abortion in legally restrictive settings.

## CONFLICT OF INTEREST STATEMENT

All authors declare no conflict of interest, including no financial relationships with any organizations that might have an interest in the submitted work in the previous three years and no other relationships or activities that could appear to have influenced the submitted work.

## ETHICS APPROVAL STATEMENT

The Institutional Review Boards of the Guttmacher Institute (IRB00002197), Johns Hopkins Bloomberg School of Public Health (IRB00008436), Addis Ababa University (075/13/SPH), Makerere University, and the Uganda National Council for Science and Technology provided ethical approval for this study.

## PATIENT CONSENT STATEMENT

Informed consent to publish anonymized data from the research was obtained from all participants recruited to the study.

## PERMISSION TO REPRODUCE MATERIAL FROM OTHER SOURCES

No material from other sources is included in this submission

## Supporting information



Appendix: Supplemental Materials

## Data Availability

The data that support the findings of this study are available on request from the corresponding author. The data are not publicly available due to privacy or ethical restrictions.

## Footnotes

1 Data collection in Uganda was paused June–early August 2021 due to COVID‐related travel restrictions.

2 Urban population estimates for 2021: Uganda = 25 percent, Ethiopia = 22 percent, accessed on April 4, 2022 from https://data.worldbank.org/indicator/SP.RUR.TOTL.ZS. World Bank urban population estimates are based on the United Nations Population Division's World Urbanization Prospects: 2018 Revision.
