# Supplementary material for: Estimating the Social Visibility of Abortions in Uganda and Ethiopia Using the Game of Contacts
Source: Stud Fam Plann. 2024 Nov 12;55(4):291–314. doi: 10.1111/sifp.12278 (PMC11636778; doi:10.1111/sifp.12278)
Supplement: Supplementary file 1 — Appendix: Supplemental Materials [file SIFP-55-291-s001.pdf]

## Appendix: Supplemental Materials

### A. Names selected for Game of Contacts

**Table A1. Selected names and name frequency, by site**

| | Uganda | | Ethiopia | |
|---|---|---|---|---|
| | Kampala | Rakai | Addis Ababa | Oromia |
| More common | Ruth<br>Christine<br>Sarah<br>Mary/Maria<br>Mariam<br>Prossy<br>Rose/Rosette<br>Florence | Florence<br>Josephine<br>Maria<br>Betty<br>Rose<br>Babirye<br>Nakato<br>Nabukenya | Tigist<br>Mekdes<br>Kidist<br>Betelhem<br>Frehiwot<br>Almaz<br>Emebet<br>Hayat | Burtukaan<br>Caaltuu<br>Daraartuu<br>Faaxumaa<br>Geexee<br>Kadijjaa<br>Tigist<br>Zaaraa |
| Somewhat common | Margaret<br>Viola<br>Lilian<br>Phiona<br>Racheal<br>Irene<br>Hellen<br>Ritah | Nansasi<br>Nabbira<br>Ruth<br>Mariam<br>Nakanaabi<br>Nambusi<br>Nabankema<br>Naluyiga | Alem<br>Aster<br>Belaynesh<br>Hayimanot<br>Helina<br>Hirut<br>Kalkidan<br>Selam | Abarruu<br>Baqqaluu<br>Ayinaalam<br>Dirribee<br>Hawwaa<br>Hiruut<br>Lalisee<br>Loomii |
| Rare | Samalie<br>Mercy<br>Claire<br>Hope<br>Martha<br>Gertrude<br>Gloria<br>Dorah | Easter<br>Egidiya<br>Enida<br>Epinezi<br>Vonita<br>Vumiliya<br>Yermani<br>Yyawe | Yimenyushal<br>Yimtubezina<br>Zehabu<br>Zemetawreke<br>Zemzem<br>Zenabeneshi<br>Zeru<br>Zewditu | Aagituu<br>Abbaayinesh<br>Adaanuu<br>Arfaase<br>Boggee<br>Eden<br>Ariitee<br>Zinaabuwaa |

## B. Game of Contacts Sensitivity Tests

*Question Order Effects*

We assessed whether game fatigue may have led to underreporting of both the number of alters as well as the proportion of alters who knew of the respondent's abortion. Because the game is played with a physical set of cards that the interviewer shuffles at the start of every interview, each name is asked in a random order for every respondent. As such, the average number of reported alters and the proportion of alters that are aware of the respondent's abortion should remain relatively constant throughout the game if fatigue was not an issue. We calculated the average number of known alters and the proportion of alters that knew of the respondent's abortion for each round of the game (name 1-name 24).

Figure B1 displays the mean number of identified in each round. In Uganda, the average number of alters reported per round remained relatively consistent throughout the game. In Ethiopia, there appears to be a slight decrease in the average number reported starting in round 9 or 10. However, this decrease is small in magnitude and does not appear to be monotonic with each additional round.

Figure B2 displays the of alters who knew of respondent's abortion in each round. There is very little variation in this proportion by game round in Uganda. While variation is observed in Ethiopia, there is no consistent pattern to the changes in the proportions. It is possible that the increased variation in Ethiopia is a result of the smaller sample size there, and it is unlikely that this variation is due to game fatigue.

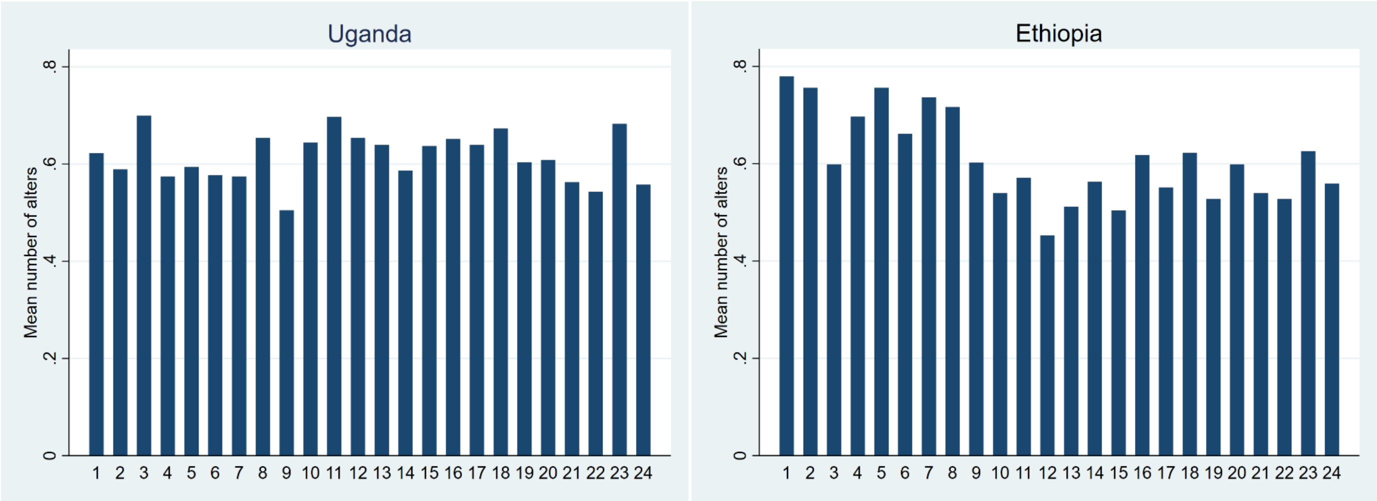


**Figure B1. Mean number of alters identified by questions order, in Uganda and Ethiopia**

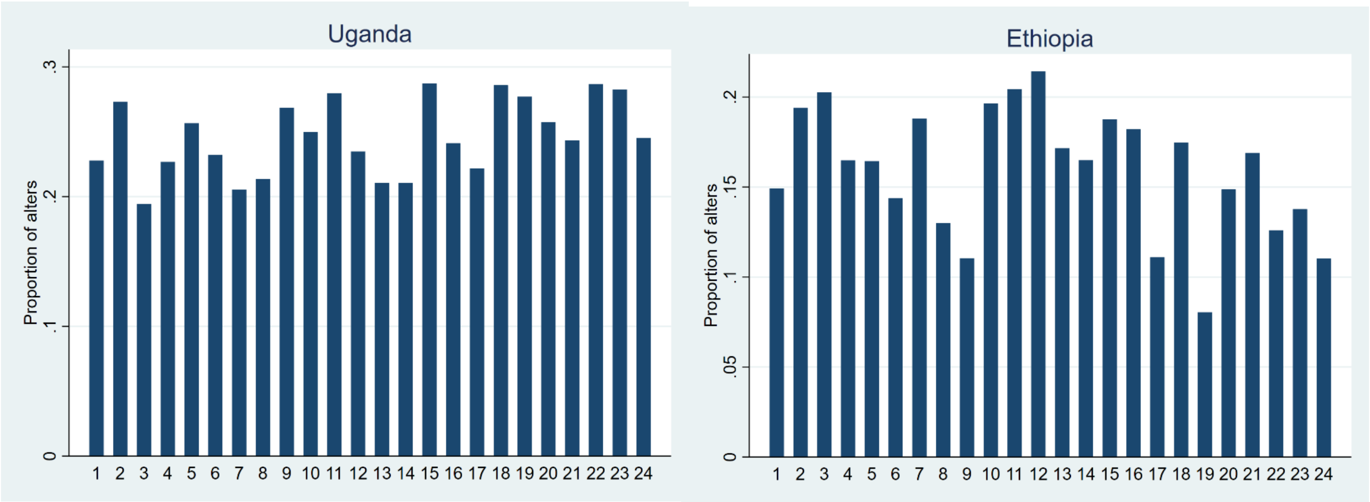


**Figure B2. Proportion of alters who knew o respondent's abortion by questions order, in Uganda and Ethiopia**

*Name effects*

The study names were chosen in order to generate a random sample of the respondent's social network members. We also assume that there is no relationship between the visibility of a respondent's abortion and the first name of her social network member. A violation to this assumption would result in name effects, where alters with a particular name are more or less likely to know about the respondent's abortion compared to alters with other names. To assess whether this bias may exist in our study, we first calculated the average number of identified alters with each name. Next, we calculated the proportion of alters with each name who knew about the respondent's abortion. We also assessed the stability of visibility estimates after iteratively dropping one name at a time.

Figure B3 displays the mean number of alters identified with each name (regardless of in which survey round that name appeared.) Because we selected names that were common, somewhat common, and rare in each setting, the observed variation in number of identified alters by name is expected.

Figure B4 displays the proportion of alters with each name who knew of the respondent's abortion. These proportions are similar across names within sites, with the exception of a few outliers. However, this is likely a function of sample size, as these outliers can be explained by the small number of alters who were identified with those names (see notes below Figure B4). These results suggest that knowledge of the respondent's abortion status is in fact independent of the alters first name.

Figure B5 displays the results of the iterative process where we dropped one name at a time and recalculated the abortion visibility estimate excluding that name. The results showed almost no variation in the abortion visibility estimate throughout this process, indicating that no individual name is not unduly biasing the results.

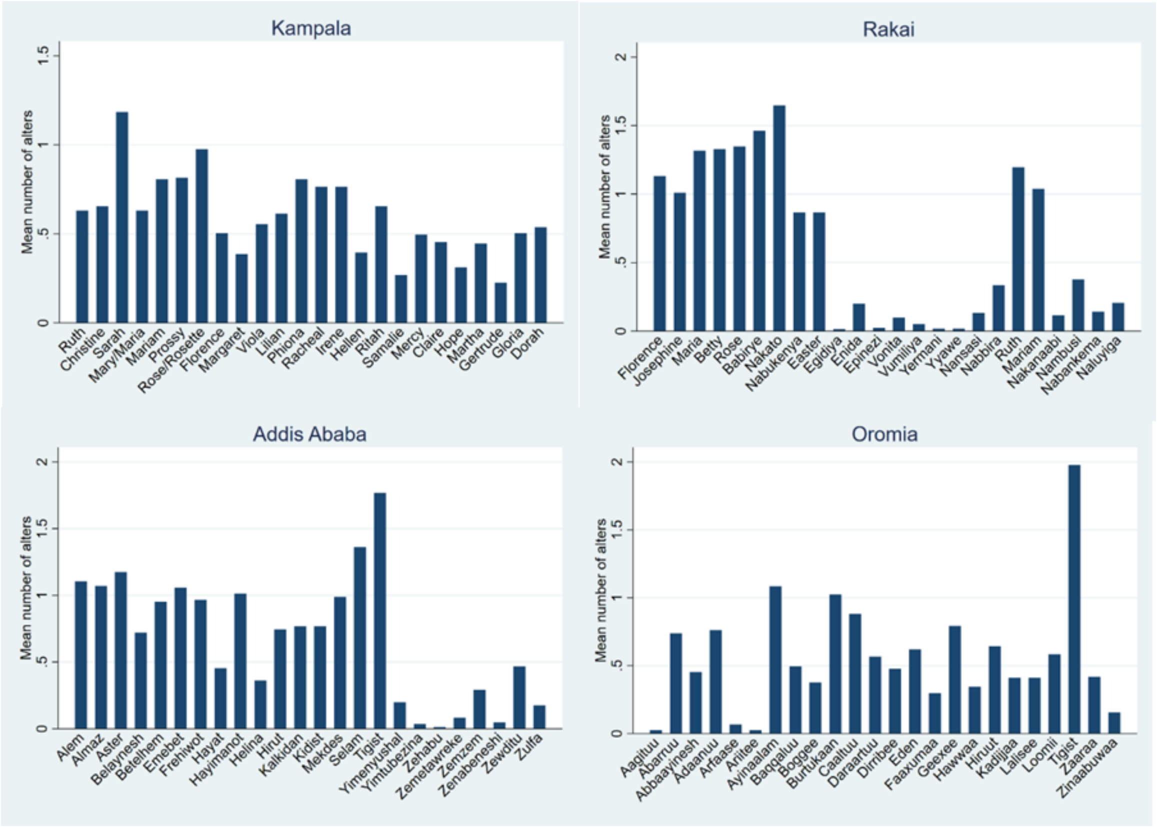


**Figure B3. Mean number of alters identified with each name, by site**

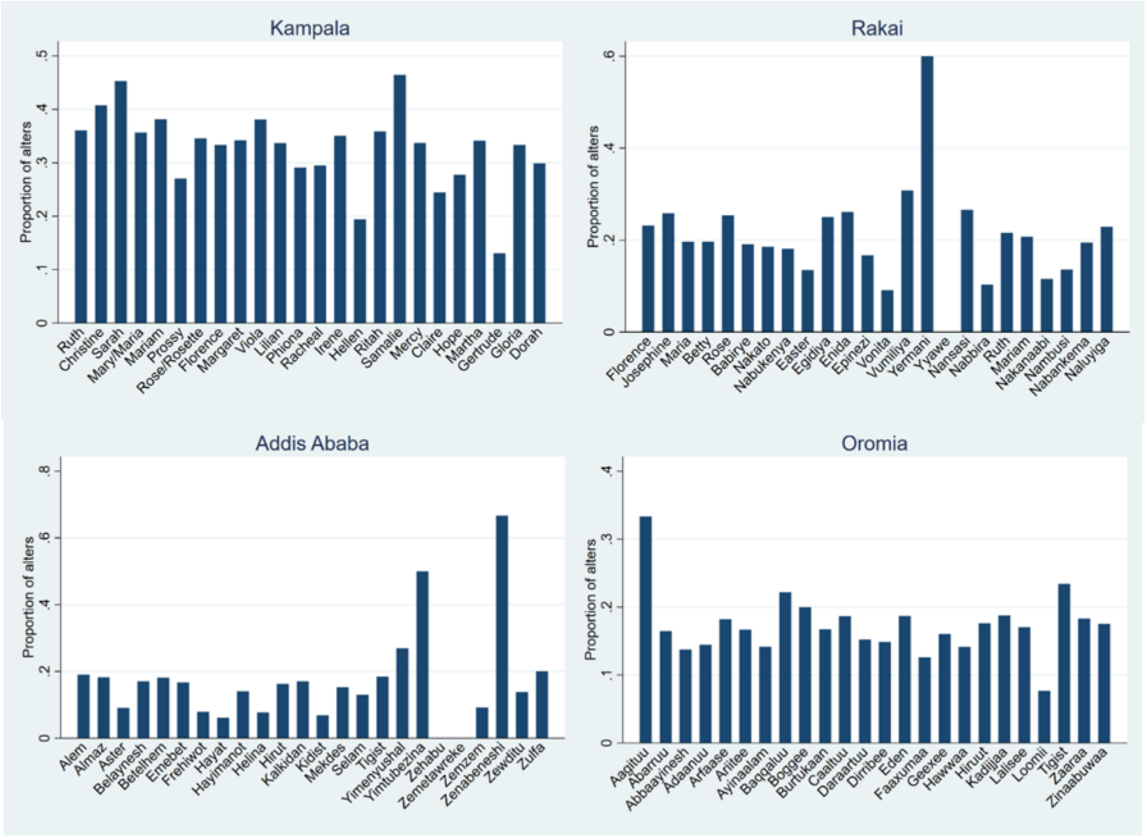


**Figure B4. Proportion of alters with each name who knew of the respondent's abortion, by site***

**Outliers: Rakai: Yermani (60%, n=5), Yyawe (0%, n=5; Oromia: Aagituu (33%, n=3); Addis Ababa: Yimtubezine (50%, n=2), Zehabu (0%, n=1), Zemetawreke (0%, n=6), Zenabeneshi (67%, n=3)*

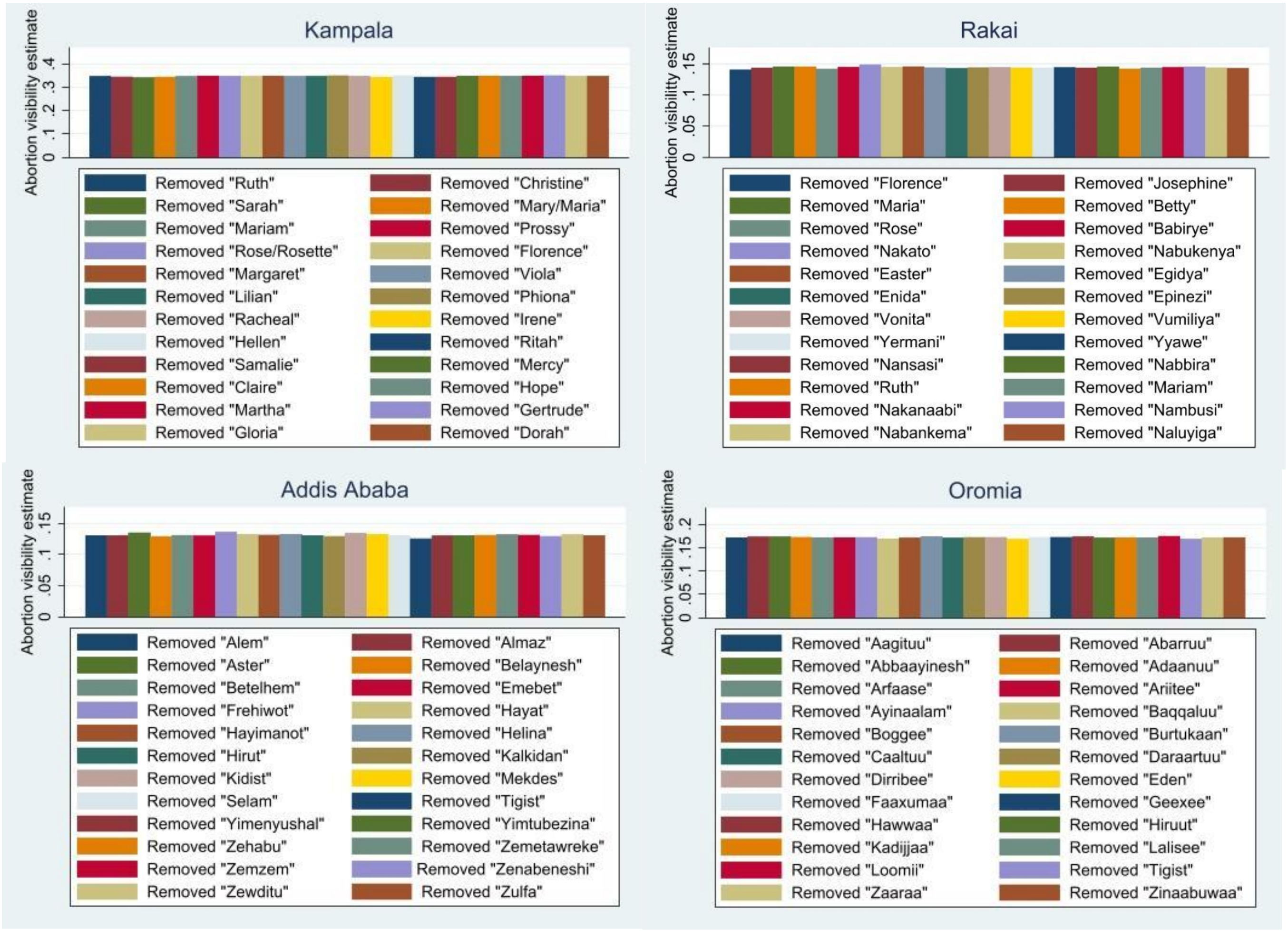


**Figure B5. Variation in abortion visibility estimates throughout iterative name-dropping process, by site**

## C. Differences in the visibility of abortions by key socioeconomic characteristics

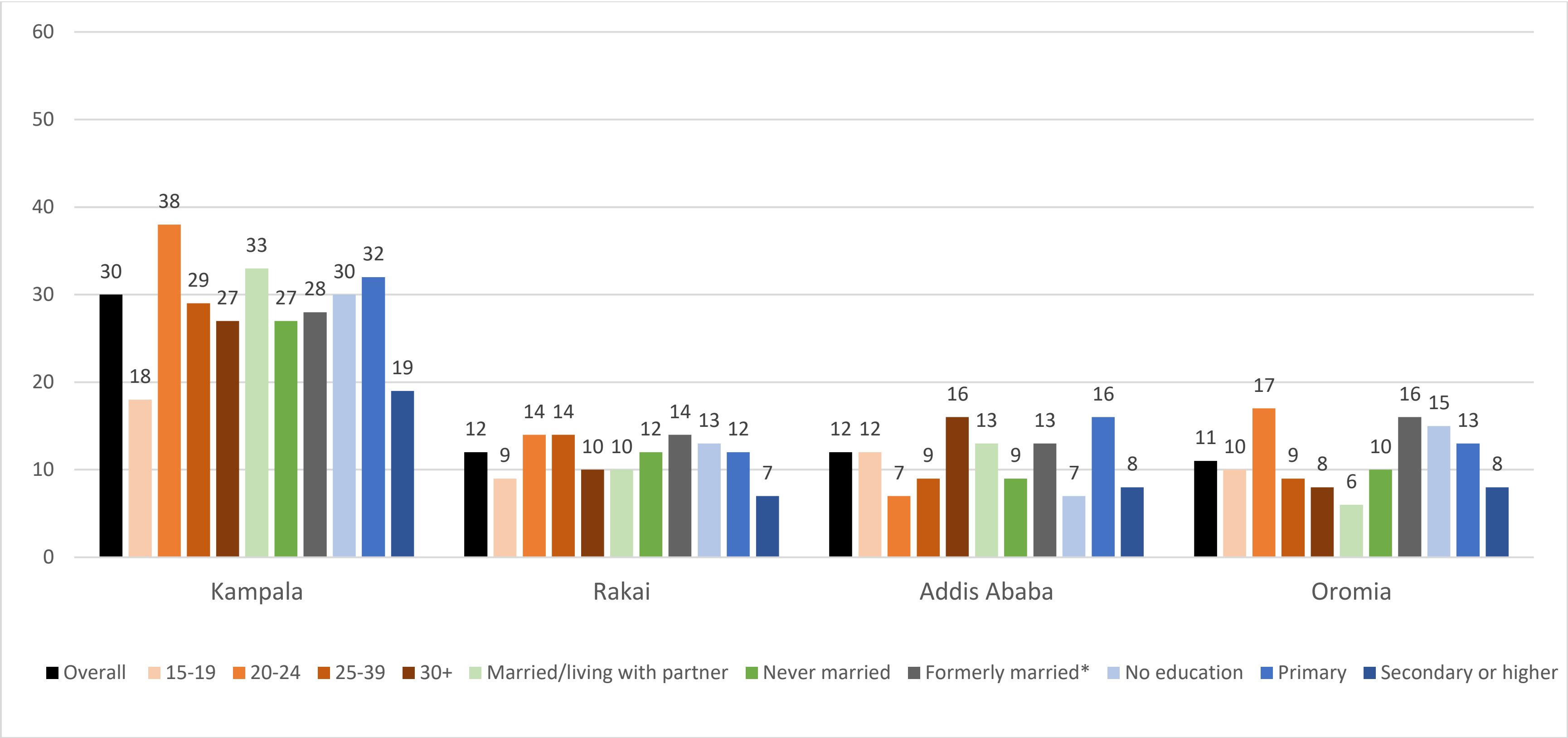


**Figure C1. Abortion visibility estimates, overall and by sociodemographic characteristics in each site**

*Formerly married = widowed or divorced
